# Rationalization of stereospecific binding of propranolol to cytochrome P450 2D6 by free energy calculations

**DOI:** 10.1007/s00249-012-0865-x

**Published:** 2012-10-20

**Authors:** Gabor Nagy, Chris Oostenbrink

**Affiliations:** Institute of Molecular Modeling and Simulation, University of Natural Resources and Life Sciences, Muthgasse 18, 1190 Vienna, Austria

**Keywords:** Hamiltonian replica exchange, CYP2D6, Stereoselectivity, Free energy calculations, Improved sampling, Thermodynamics

## Abstract

**Abstract:**

Cytochrome P450 2D6 is a major drug-metabolising enzyme with a wide substrate range. A single-point mutation introduced in this enzyme induces stereoselective binding of *R* and *S*-propranolol whereas the wild type has no preference. The system has previously been studied both experimentally and computationally (de Graaf et al. in Eur Biophys J 36:589–599, 2007a). The in silico study reported hysteresis and significant deviations from closure of thermodynamic cycles, probably because of lack of sampling. Here, we focus on the effect of prolonged simulation time and enhanced sampling methods, such as Hamiltonian replica exchange, to reduce these problems and to improve the precision of free energy calculations. Finally we rationalize the results at a molecular level and compare data with experimental findings and previously estimated free energies.

**Graphical Abstract:**

Propranolol (PPD) binds stereospecifically to cytochrome P450 2D6, if the mutation F483A is introduced, by reducing binding affinity to *R*-PPD. This observation has previously been studied by molecular dynamics, by use of several different thermodynamic cycles. Previous computational results suffered from internal inconsistencies, probably because of insufficient sampling. Here we use prolonged simulation time and Hamiltonian replica exchange to overcome the inconsistencies, and give a possible rationalization of the phenomenon at a molecular level.
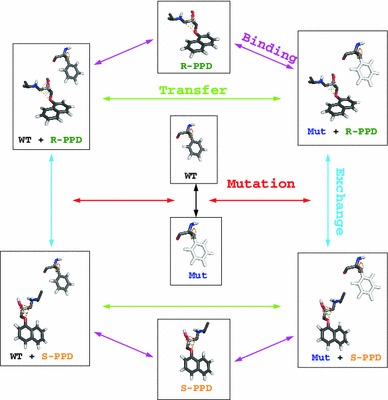

## Introduction

The cytochrome P450s (CYPs) are a superfamily of heme containing oxido-reductase enzymes; because of their wide substrate range they are of crucial importance in the metabolism of a variety of compounds. Cytochrome P450s are present in all five biological kingdoms, and are particularly important in the metabolism of drugs administered to humans. CYP2D6, the second most important Cytochrome P450, is involved in the metabolism of more than 15 % of commercial pharmaceuticals (Rendic 2002; van Waterschoot et al. 2006; Pelkonen et al. 2008). In addition to this, its genetic diversity highlights the importance of understanding metabolism by this enzyme, as well as its inhibition (Ito et al. 2008). The function of various active site residues in the binding and metabolic profiles of substrates and inhibitors has been thoroughly studied (de Graaf et al. 2007b). One of these studies (Lussenburg et al. 2005) revealed that the F483A mutation may change substrate specificity and regioselectivity. This particular mutation induced stereoselectivity of propranolol (PPD) binding, because the binding affinity of *R*-PPD was reduced by a factor of 20 in the mutant whereas the affinity of *S*-PPD remained similar to the affinity in the wild type enzyme. This experimental find was followed by computational studies by de Graaf et al. (2007a) which suggested use of four different approaches to estimate the stereoselectivity of CYP2D6 and its mutant F483A toward propranolol. Although some of the results in this paper were in very good agreement with experimentally determined free energies, the paper also reported internal inconsistencies in the form of different free energies for forward and reverse processes (hysteresis) and a failure to close thermodynamic cycles, along which the free energy estimates should add up to zero. The simulations were started from a homology model of the enzyme, and were relatively short, suggesting insufficient sampling of the conformational space. In accordance with their wide substrate specificity CYPs are known to have remarkable flexibility and malleability in their active sites (Guengerich 2006; Oostenbrink et al. 2012). The only available X-ray structure of CYP2D6 at the start of this work (pdb code 2F9Q) was an apo structure in which the active site was relatively small (Rowland et al. 2006). It was, therefore, not suitable for explaining the metabolism of many substrates, whereas the homology model (de Graaf et al. 2007b) or structures derived from careful molecular dynamics (MD) simulations (Hritz et al. 2008) may be more appropriate for the task. Other examples are available in papers by Kjellander et al. (2007) and Rydberg and Olsen (2012). Recently, holo structures of CYP2D6 with different ligands have become available (pdb codes 3QM4, 3TMB, 3TDA); these confirm the dynamic behaviour of F483 observed in MD simulations (Hritz et al. 2008). Note that the latter work also implicated F483 as being crucial to understanding the binding of substrates. The flexibility and malleability of the active site are also significant challenges to accurate free energy calculations, which depend on sufficient sampling of the conformational space to capture all energy and entropy contributions (Beveridge and Dicapua 1989; Kollman 1993; Chipot and Pearlman 2002).

Here we will rebuild the original thermodynamic cycles of CYP2D6 wild type and F483A mutant enzymes with PPD enantiomers, on the basis of the available crystallographic structure (Rowland et al. 2006). By using prolonged simulation times and advanced sampling methods—i.e. Hamiltonian replica exchange—we improve the precision of the reported free energy calculations.

## Methods

All molecular dynamics simulations were carried out using the GROMOS11 package for molecular simulations (Riniker et al. 2011; Schmid et al. 2012). Molecular interactions were described according to GROMOS force field parameter set 45A4 (Lins and Hünenberger 2005; the slightly different 43A1 (Daura et al. 1998) set was used in the original approach). Initial coordinates were derived from the crystal structure of CYP2D6 (pdb 2F9Q). Note that the N-terminal membrane anchor of 33 amino acids is missing from this model. Propranolol was carefully modelled into the active site by means of gradual molecular insertion, as described elsewhere (Hritz et al. 2008). Free energy calculations were performed by using thermodynamic integration (TI) (Kirkwood 1935), and Hamiltonian replica exchange (HRE; Sugita et al. 2000; Fukunishi et al. 2002; Woods et al. 2003). Visualisation was done by using PyMol (Schrödinger 2009) molecular graphics systems version 1.2r.2.

### Simulation setup

The CYP2D6 wild type enzyme with *R*-propranolol in the binding site was solvated in a rectangular box containing 17019 simple point charge (SPC) (Berendsen et al. 1981) water molecules and 5 Na^+^ ions. Individual CYP2D6 and *R*-propranolol structures were simulated after solvation in cubic SPC water boxes (containing 16,659 water molecules and 6 Na^+^ ions, and 1,812 water molecules, respectively). The systems were equilibrated and heated from 60 to 298 K while gradually removing the positional restraints on the solute atoms (initial force constant 2.5 × 10^4^ kJ/mol/nm^2^) in five discrete simulations of 20 ps. All simulations were performed under periodic boundary conditions, using a 2-fs time step and the SHAKE algorithm (Ryckaert et al. 1977) to constrain bond lengths and H–O–H bond angles. The weak-coupling algorithm (Berendsen et al. 1984) was used to maintain constant temperature and pressure, with relaxation times of 0.1 and 0.5 ps, respectively. Overall translational and rotational motion was removed every 1,000 steps, and for long-range interactions the reaction field method (Tironi et al. 1995) was used with a 1.4 nm cutoff and 61 as dielectric permittivity (Heinz et al. 2001). Error estimates were calculated from block averages (Allen and Tildesley 1989).

### Thermodynamic integration

TI simulations are widely accepted standard methods used to obtain free energies for alchemical changes in equilibrium molecular dynamics (Kirkwood 1935; Kollman 1993). A (virtual) reaction coordinate (*λ*) between two physically meaningful end states is defined, and a series of MD simulations is started from an initial state via discrete intermediate values of *λ*, until the other end state is reached. At each individual point along the path, the ensemble average of the derivative of the Hamiltonian (*H*) with respect to *λ* is determined, and the free energy difference (Δ*G*) is obtained by integrating over the derivatives along the path, as in Eq. 1:

1 $$ \Updelta G = \int\limits_{0}^{1} {\left\langle {\frac{{\partial {\rm H}}}{\partial \lambda }} \right\rangle_{\lambda } {\text{d}}\lambda } $$

**Fig. 1:**
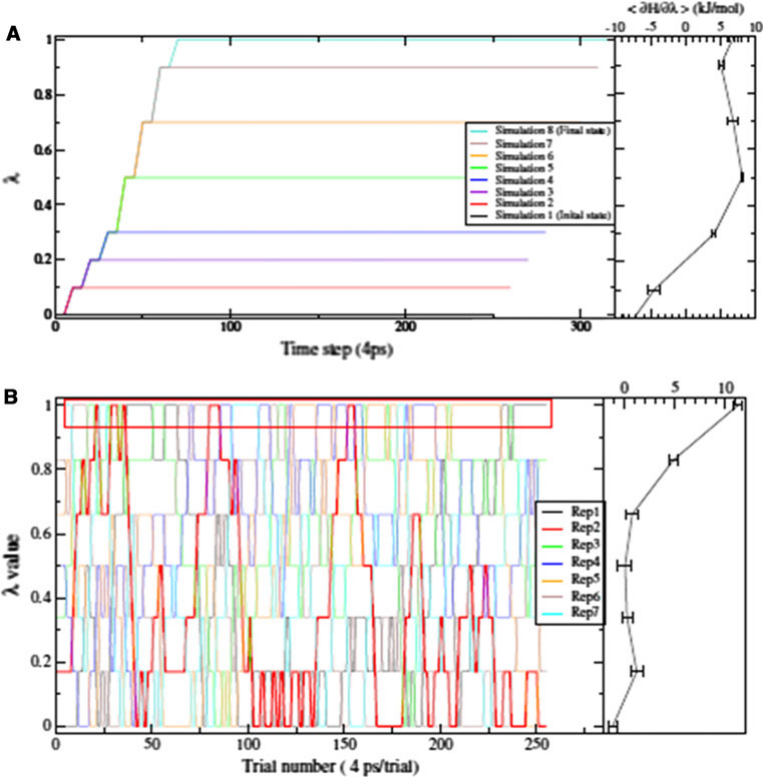
Schematic representation of the free energy methods used here. **a** Thermodynamic integration simulations: *on the left, different colours* represent the individual MD simulations started sequentially at different *λ* points; *on the right* the $\partial {\rm H}/\partial \lambda$ ensemble average and uncertainty is shown (free energy profile); these are used for the integration. **b** The Hamiltonian replica exchange simulations. The different replicas are started simultaneously and are allowed to exchange between *λ* points to obtain a better ensemble average. Replica 2 started from *λ* point 0.166 is marked with a *thicker line* to show how it visits all intermediate states. The *boxed* trajectory from different replicas was used to calculate the ensemble average for *λ* = 1.0

Because free energy is a state function, the free energy difference is completely defined by the two end states and not by the intermediates. However, obtaining the correct free energy value requires properly converged ensemble averages at each *λ*. A schematic depiction of TI can be seen in Fig. 1a. In an ideal simulation with infinite simulation time, the free energy obtained is independent from the end state from which the simulation is initiated, but this might not be true in practice. Because TI simulations are commonly started from conformations obtained at previous *λ* points (after some equilibration), energy barriers might lead to very different sampling of conformational space—depending on the starting structure—leading to different calculated free energies. The difference between the free energy estimates for the forward and backward processes is called hysteresis.

Here, two series of TI simulations were carried out for each individual change (see the section “Simulated processes”, below). Preliminary TI simulations were performed with 120 ps simulation time per *λ* point. These simulations enabled us to determine the initial hysteresis and deviations from cycle closure. Furthermore, it was also used to determine whether additional *λ* points are required to obtain a smooth free energy profile, and to generate starting coordinates for the main TI and HRE simulations. The preliminary TI simulations were started from the last frame of a 1-ns classical MD simulation of the wild type enzyme with *R*-PPD, or from the equilibrated structures of either *R*-PPD or wild type CYP2D6. The main TI runs were started as prolongation of the preliminary runs, however, slight modifications were introduced (see below). Each prolonged TI simulation was performed for 1 ns per *λ* point and was evaluated independently from the preliminary simulation.

### Hamiltonian replica exchange

Hamiltonian replica exchange TI methods share principles with TI, but all MD simulations for different states (the replicas) are started independently, and simultaneously. At given time periods (here 4 ps), neighbouring replicas are allowed to exchange their *λ* values, following the Metropolis criterion (Sugita et al. 2000; Woods et al. 2003). A schematic representation of the HRE process is shown in Fig. 1b. This approach helps the individual replicas to visit hard-to-reach parts of the conformational space, should an energy barrier exist in some of the states only (Woods et al. 2003; Riniker et al. 2011; Steiner et al. 2011). After the simulations are finished, the trajectory of each replica is sorted according to *λ*—thus obtaining an ensemble average at each *λ* point—and the free energy of the process is determined with the same integration as described above (Eq. 1). The possibility of exchanging coordinates between *λ* values should improve conformational sampling at each *λ* point, leading to a more precise free energy profile, and better convergence of the calculated free energy differences.

All HRE simulations were performed using the same parameters as the main TI simulations, for 1 ns over all *λ* values. Because of hardware limitations the F483A mutations could only be performed using 12 replicas (and *λ* points) instead of the 16 *λ* points of TI. The number of *λ* points for the PPD inversions was not changed, but the *λ* points were slightly shifted for the HRE simulation to promote exchange probabilities in regions where exchange was relatively seldom. Replica exchange starting coordinates were taken from the preliminary TI simulations of the appropriate *λ* points.

### Simulated processes

Two types of alchemical change were introduced: mutation of phenylalanine 483 into alanine and inversion of propranolol from the *R* configuration to the *S* configuration. For the TI calculations the reverse processes were also performed, using the last frame of the forward process as the starting coordinates. For replica exchange TI simulations—as the directionality of the process disappears—a single simulation per process was performed.

#### F483A mutations

**Fig. 2:**
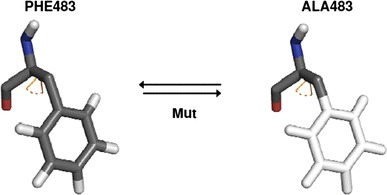
Phenylalanine mutation process. Stick representation of phenylalanine 483 and its change to alanine. Carbon atoms represented in *dark gray*, hydrogens in *light gray*, oxygen in *red*, nitrogen in *blue*, and dummy atoms in *white*. The side chain torsion, *χ*_1_, is depicted in orange. CB was changed from a CH_2_ group in F483 into a CH_3_ in A483

The F483 mutation in CYP2D6 was simulated by changing atoms of the phenyl ring into non-interacting dummy atoms and turning the CH_2_ group of the beta carbon into a CH_3_ group, as depicted in Fig. 2. To smoothen molecular changes, soft core potentials (Beutler et al. 1994) were used with soft core values of 0.5 nm^2^ for electrostatic interactions and 0.5 for the Van der Waals interactions. Mutations of F483A are abbreviated as Mut, Mut_R, Mut_F, and Mut_S, indicating mutation processes in the absence of PPD, with *R*-PPD, with a planar PPD intermediate, or with *S*-PPD bound, respectively. After the preliminary TI simulations, five additional (to the initial 11) *λ* points were inserted for mutations, to make the integration curve smoother. Mutation type processes now consist of 16 simulations for TI at *λ* points 0.0, 0.05, 0.1, 0.15, 0.2, 0.3, 0.4, 0.5, 0.6, 0.7, 0.8, 0.85, 0.9, 0.93, 0.95, and 1.0; and 12 simulations for HRE (not including 0.05, 0.15, 0.93, 0.95).

#### PPD inversions

**Fig. 3:**
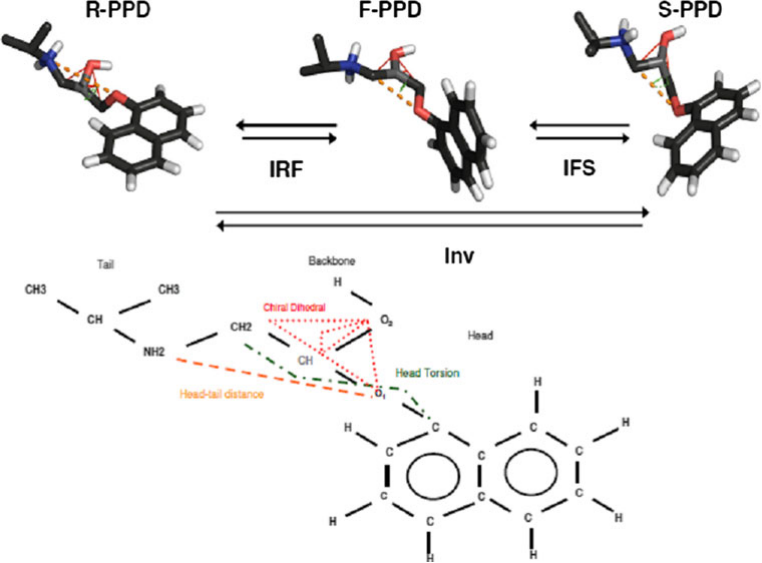
Propranolol inversion. Stick representation and topological model of inversion of propranolol via a planar intermediate. Carbon atoms represented in *dark gray*, hydrogens in *light gray*, oxygen in *red*, and nitrogen in *blue*. The head–tail distance is represented by a *dashed orange line*, the head torsion by a *green dot*-*dash line*, and the chiral improper dihedral angle by a *red dotted line*

As the chiral CH group is described by a single atom in the GROMOS 45A4 united atom force field the inversion type process involves solely the improper dihedral angle on the chiral centre (depicted in a lighter shade in Fig. 3). The zero improper dihedral energy angle was changed from +35.26° (*R*-PPD) to −35.26° (*S*-PPD). The change was introduced in two steps, first by changing the *R*-PPD into a planar intermediate (called F-PPD), then changing the intermediate state into *S*-PPD. The bond angles around the chiral centre were also increased to 120° in the F-PPD state. Because it was found in simulations of PPD in water that the head torsion was not sampled properly, the force constant of this covalent dihedral angle interaction was reduced to 0 kJ/mol for the planar intermediate state of the molecule. A representation of the PPD inversion defining the head torsion is depicted in Fig. 3. Inversion processes are named IRF, IFS, or Inv for conversion from *R*-PPD to F-PPD, F-PPD to *S*-PPD, and for full inversion, respectively, followed by _wt or _M if the process takes place inside a wild type or mutant CYP2D6. Inversions were done using 2 × 7 *λ* points for both TI (0.0, 0.1, 0.3, 0.5, 0.7, 0.9, 1.0) and HRE (0.0, 0.16, 0.34, 0.5, 0.66, 0.83, 1.0) simulations.

## Results and discussion

**Fig. 4:**
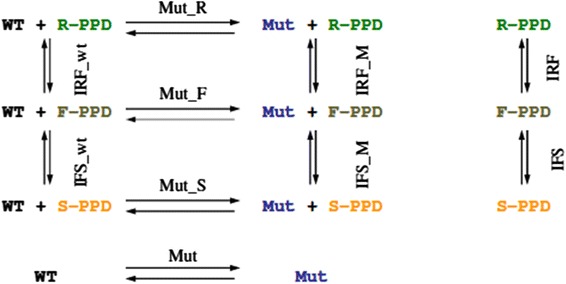
Thermodynamic scheme. End states are connected by *arrows*, where *WT* is wild type CYP2D6, *Mut* is CYP2D6 mutant F483A, *R-PPD* is *R*-propranolol, *S-PPD* is *S*-propranolol, and *F-PPD* is the planar intermediate. *Each arrow* represents a thermodynamic integration simulation, which was used to build the scheme starting from the WT + *R*-PPD state. WT and *R*-PPD models were made by removing the appropriate molecules from the WT + *R*-PPD model

De Graaf et al. described four different approaches with different thermodynamic cycles, which were handled separately, and could directly be compared to the relative binding free energies derived from the dissociation constants of spectral titrations. Here, we also calculate these free energies, and join the free energy differences into a single, thermodynamic scheme in Fig. 4. This enables us to monitor the hysteresis and deviations from cycle closure of our simulations. Additionally, the free energies of all processes were calculated by using an increasing amount of simulation time to monitor the evolution of free energies, hysteresis, and deviation from cycle closure. Finally, the trajectories from all simulations that represent the physically meaningful end states of the different processes were collected and analysed together, to obtain differences in the average behaviour of the end states and to find a structural rationalization of the hysteresis in our systems.

### Rebuilding the cycles

The thermodynamic scheme in Fig. 4 was gradually built along the various simulation arrows, starting from the CYP2D6 wild type and *R*-PPD state (WT + *R*-PPD). The integrated free energies of all processes, calculated from forward and reverse TI simulations and HRE TI after 1 ns are shown in Table 1. From these integrated free energies we calculated a number of derived quantities in Table 2, as described below:

**Table 1:** Primary free energy data

Process	TI forward (kJ/mol)	TI reverse (kJ/mol)	HRE (kJ/mol)
Δ*G*_Mut_	19.7 ± 2.4	−0.7 ± 3.2	8.8 ± 2.9
Δ*G*_Mut_R_	23.1 ± 3.3	−20.6 ± 3.3	24.4 ± 2.3
Δ*G*_Mut_F_	29.3 ± 1.6	−10.0 ± 2.6	–
Δ*G*_Mut_S_	18.1 ± 2.2	−17.0 ± 2.5	21.2 ± 2.2
Δ*G*_IRF_	−9.1 ± 0.3	8.9 ± 0.5	−8.9 ± 0.3
Δ*G*_IFS_	8.6 ± 0.5	−8.8 ± 0.5	8.6 ± 0.3
Δ*G*_IRF_wt_	−1.1 ± 0.7	3.8 ± 0.8	−2.1 ± 0.6
Δ*G*_IFS_wt_	14.4 ± 1.3	−17.8 ± 1.1	9.2 ± 0.9
Δ*G*_IRF_M_	−4.6 ± 0.7	4.7 ± 0.8	−6.5 ± 0.7
Δ*G*_IFS_M_	7.6 ± 0.9	−4.4 ± 0.8	8.0 ± 0.7

Free energy differences for all processes of the thermodynamic scheme in Fig. 4 for forward (Δ*G* ^for^) and reverse (Δ*G* ^rev^) TI and Hamiltonian replica exchange (Δ*G* ^hre^) schemes, and their statistical uncertainty after 1 ns per *λ* point simulation time

**Table 2:** Derived thermodynamic properties

Property	TI forward (kJ/mol)	TI reverse (kJ/mol)	HRE (kJ/mol)
Δ*G*_Inv_	0.5 ± 0.6	0.1 ± 0.7	0.3 ± 0.5
Δ*G*_Inv_wt_	13.3 ± 1.5	14.0 ± 1.4	7.1 ± 1.1
Δ*G*_Inv_M_	3.0 ± 1.1	−0.3 ± 1.1	1.5 ± 1.0
ΔΔ*G*_bind_^WT^	13.8 ± 1.6	14.0 ± 1.5	7.5 ± 1.2
ΔΔ*G*_bind_^Mut^	3.5 ± 1.2	−0.2 ± 1.3	1.8 ± 1.1
Δ*G*_transfer_	−10.3 ± 1.8	−14.2 ± 1.8	−5.7 ± 1.5
ΔΔ*G*_mut_^R^	3.3 ± 4.0	19.9 ± 4.6	15.5 ± 3.7
ΔΔ*G*_mut_^S^	−1.6 ± 3.3	16.3 ± 4.0	12.3 ± 3.6
ΔΔ*G*_exchange_	−5.0 ± 3.9	−3.6 ± 4.1	−3.2 ± 3.2
Δ*G*_cycle_	5.3 ± 4.3	10.6 ± 4.5	2.5 ± 3.6
ΔΔ*G*_affinity_	−7.6 ± 2.9	−8.9 ± 4.5	−4.4 ± 3.6

Calculated directly from Table 1. Quantities were calculated for forward (Δ*G* ^for^) and reverse (Δ*G* ^rev^) TI, and Hamiltonian replica exchange (Δ*G* ^hre^) schemes

#### Inversions

The inversions are calculated on the basis of Eq. 2, where _wt and _M may be added to the index to represent the inversion bound to wild type or mutant CYP2D6.

2 $$ \Updelta G_{\text{Inv}} = \Updelta G_{\text{IRF}} + \Updelta G_{\text{IFS}} $$

The value of Δ*G*_Inv_ corresponds to the free energy difference between *R*-PPD to *S*-PPD in aqueous solution. The inversion in water is an important inner standard, because in an achiral environment the free energy of the PPD inversion should be 0 kJ/mol. This criterion is met in both methods within the uncertainty ranges. The requirement ΔG_Inv_ ~ 0 kJ/mol was also met in the preliminary TI calculations, but the head torsion (Fig. 3) of PPD had a very different distribution from independent simulations of the F-PPD state (data not shown). This indicated insufficient sampling, for which the barrier of this torsion was removed in F-PPD for the remaining simulations.

#### Cycle closure

The free energy change along the main cycle should lead to a value of 0 kJ/mol exactly, as free energy is a state function. The different estimates of the cycle closure were calculated as follows (Fig. 4):

3 $$ \Updelta G_{\text{cycle}}^{\text{for}} = \Updelta G_{{{\text{Inv\_wt}}}}^{\text{for}} + \Updelta G_{{{\text{Mut\_S}}}}^{\text{for}} + \Updelta G_{{{\text{Inv\_M}}}}^{\text{rev}} + \Updelta G_{{{\text{Mut\_R}}}}^{\text{rev}} $$

4 $$ \Updelta G_{\text{cycle}}^{\text{rev}} = \Updelta G_{{{\text{Mut\_R}}}}^{\text{for}} + \Updelta G_{{{\text{Inv\_S}}}}^{\text{for}} + \Updelta G_{{{\text{Mut\_S}}}}^{\text{rev}} + \Updelta G_{{{\text{Inv\_wt}}}}^{\text{rev}} $$

5 $$ \Updelta G_{\text{cycle}}^{\text{hre}} = \Updelta G_{{{\text{Inv\_wt}}}}^{\text{hre}} + \Updelta G_{{{\text{Mut\_S}}}}^{\text{hre}} - \Updelta G_{{{\text{Inv\_M}}}}^{\text{hre}} - \Updelta G_{{{\text{Mut\_R}}}}^{\text{hre}} $$

For the preliminary TI runs the average Δ*G*_cycle_ was more than 20 kJ/mol; in the prolonged TI cycle only 8 kJ/mol remains whereas for the replica exchange cycle 2.5 kJ/mol was calculated. Because the uncertainty for cycle closures is approximately 4 kJ/mol, the cycles did not close in the TI calculations.

#### Hysteresis

Hysteresis is defined as the difference between the free energies associated with the forward and reverse simulation of a process. By definition, hysteresis is not observed for the HRE simulations. For TI simulations the hysteresis can be calculated directly from Table 1. Taking the uncertainties into account only 5 of the 10 processes are free from hysteresis, after 1 ns of simulation at every *λ* point. The sum of the absolute hysteresis of all processes is calculated here as:

6 $$ \Updelta G_{\text{hysteresis}} = \sum {\left| {\Updelta G^{\text{for}} + \Updelta G^{\text{rev}} } \right|} $$

Δ*G*_hysteresis_ amounts to ~50 kJ/mol, of which 74 % can be attributed to the Mut and Mut_F processes, which are not part of the main cycle.

#### Approach 1: relative binding free energy differences

The difference between *R*-PPD and *S*-PPD binding to a CYP2D6 enzyme—calculated with a thermodynamic cycle from the inversion of PPD in water and in the appropriate protein—can be estimated by use of Eqs. 7 and 8.

7 $$ \Updelta \Updelta G_{\text{bind}}^{\text{WT}} = \Updelta G_{{{\text{Inv\_wt}}}} - \Updelta G_{\text{Inv}} $$

8 $$ \Updelta \Updelta G_{\text{bind}}^{\text{Mut}} = \Updelta G_{{{\text{Inv\_M}}}} - \Updelta G_{\text{Inv}} $$

Because the wild type CYP2D6 shows no stereoselectivity (de Graaf et al. 2007a) we expect a binding free energy difference near 0 kJ/mol for the wild type enzyme. Similarly, in the mutant *S*-PPD binds more strongly, suggesting a more negative value for $\Updelta \Updelta G_{\text{bind}}^{\text{Mut}}$. However, in agreement with the previous computational results (de Graaf et al. 2007a), our calculations show binding is less favourable for *S*-PPD in the wild type (positive $\Updelta \Updelta G_{\text{bind}}^{\text{WT}}$) and that there is almost no difference within the mutant.

#### Approach 2: propranolol transfer free energy difference

The relative difference of free energies of transfer of *R*-PPD and *S*-PPD from the CYP2D6 wild type to the mutant can be calculated with a thermodynamic cycle from the inversion free energies within the proteins, and is identical with the differences of ΔΔ*G*_bind_.

9 $$ \Updelta \Updelta G_{\text{transfer}} = \Updelta G_{{{\text{Inv\_M}}}} - \Updelta G_{{{\text{Inv\_wt}}}} = \Updelta \Updelta G_{\text{bind}}^{\text{Mut}} - \Updelta \Updelta G_{\text{bind}}^{\text{WT}} $$

#### Approach 3: the effect of PPD on F483A mutation

The presence or absence of PPD modifies the free energy of mutation. This effect can be calculated from the free energy of mutation without PPD and with either *R*-PPD or *S*-PPD.

10 $$ \Updelta \Updelta G_{\text{mut}}^{\text{R}} = \Updelta G_{{{\text{Mut\_R}}}} - \Updelta G_{\text{Mut}} $$

11 $$ \Updelta \Updelta G_{\text{mut}}^{\text{S}} = \Updelta G_{{{\text{Mut\_S}}}} - \Updelta G_{\text{Mut}} $$

#### Approach 4: PPD exchange between CYP2D6 forms

The relative free energy difference of exchanging *R*-PPD with *S*-PPD in the wild type and mutant CYP2D6 can be calculated from mutation processes with *R*-PPD and *S*-PPD, and is identical with differences of ΔΔ*G*_mut_.

12 $$ \Updelta \Updelta G_{\text{exchange}} = \Updelta G_{{{\text{Mut\_S}}}} - \Updelta G_{{{\text{Mut\_R}}}} = \Updelta 
\Updelta G_{\text{mut}}^{\text{S}} - \Updelta \Updelta G_{\text{mut}}^{\text{R}} $$

Although the different approaches yield different relative free energies, ΔΔ*G*_exchange_ (approach 4) and ΔΔ*G*_transfer_ (approach 2) should be equal if the main thermodynamic cycle closes. The original values amount to 4.9 and −7.5 kJ/mol respectively (de Graaf et al. 2007a), and we calculated −4.3 and −12.3 kJ/mol for the TI cycles (averaged over forward and reverse cycles), and −3.2 and −5.7 kJ/mol for the Hamiltonian replica exchange calculations. The obvious improvement of the relative free energies is that both approaches now point in the same direction, in contrast to the original two values. The value of ΔΔ*G*_exchange_ and ΔΔ*G*_transfer_ derived from experimental values is −7.7 kJ/mol. If we average the two relative free energies using Eq. 13, we obtain the relative affinity difference of wild type and mutant CYP2D6.

13 $$ \Updelta \Updelta G_{\text{affinity}} = (\Updelta \Updelta G_{\text{exchange}} + \Updelta \Updelta G_{\text{transfer}} )/2 $$

The calculated value ΔΔ*G*_affinity_ is −8.3 and −4.5 kJ/mol for TI and HRE calculations respectively, which suggest that replica exchange slightly underestimates the free energies whereas TI results in significant scatter of the values (individual forward and reverse estimates ranging from −3.6 to −14.2 kJ/mol).

### Evolution of thermodynamics

To evaluate the effect of simulation time length on convergence of the free energy differences, the summed absolute hysteresis for the TI simulations (Eq. 6), and Δ*G*_cycle_ (Eqs. 3–5) and ΔΔ*G*_affinity_ (Eq. 13) for both TI and HRE cycles were calculated after increments of 200 ps of simulation time. The results of our calculations are shown in Table 3. As expected, the uncertainty in the quantities obtained decreases slightly with increased simulation time. Surprisingly, cycle closure was already much improved in the HRE calculations after only 200 ps (the increase at 1 ns is still well within the uncertainty) whereas in the TI scheme both hysteresis and Δ*G*_cycle_ gradually decrease as sampling improves. Interestingly, ΔΔ*G*_transfer_ for TI and HRE simulation differ significantly, but both values decrease over time.

**Table 3:** Thermodynamic changes with increased simulation time

Method	Thermodynamic integration			Hamiltonian replica exchange	
Time (ps/LP)	Δ*G*_hysteresis_ (kJ/mol)	Δ*G*_cycle_ (kJ/mol)	ΔΔ*G*_affinity_ (kJ/mol)	Δ*G*_cycle_ (kJ/mol)	ΔΔ*G*_affinity_ (kJ/mol)
200.0	68.4 ± 7.5	9.7 ± 4.5	−3.7 ± 4.5	0.9 ± 4.6	−3.4 ± 4.6
400.0	58.5 ± 9.4	8.0 ± 7.6	−6.6 ± 5.4	0.1 ± 4.2	−2.9 ± 4.2
600.0	55.6 ± 8.7	7.2 ± 6.9	−7.6 ± 4.9	1.1 ± 4.2	−4.3 ± 4.2
800.0	53.6 ± 8.3	6.8 ± 6.4	−8.2 ± 4.5	0.4 ± 3.8	−4.8 ± 3.8
1,000.0	51.8 ± 8.1	8.0 ± 6.2	−8.0 ± 4.4	2.5 ± 3.6	−4.4 ± 3.6

Summed hysteresis for TI (Δ*G*_hysteresis_), averaged main cycle closure (Δ*G*_cycle_), and relative affinity difference between wild type and mutant CYP2D6 (ΔΔ*G*_affinity_), calculated from the ensemble averages after a given simulation time length per *λ* point (ps/LP)

**Fig. 5:**
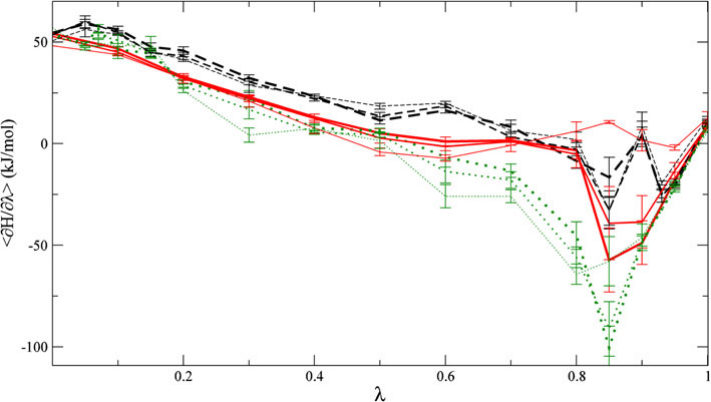
Time evolution of free energy profiles. Free energy profiles for forward (*black dashed lines*), inverted reverse (*dotted green lines*) thermodynamic integration, and Hamiltonian replica exchange (*red solid lines*) simulations of the mutation process in absence of PPD (Mut). The *thickness of the curves* represents simulation length (*thin* 200, *intermediate* 600, *thick* 1,000 ps/*λ* point)

The effect of simulation time on individual processes was also investigated. In many cases, forward TI simulations seemed better converged than their reverse counterparts, especially for the mutation type processes, where the PHE483 side chain needs to find the proper orientation. For the replica exchange simulations, the free energy differences are not so dependent on the simulation time, but are somewhat higher than either of the TI simulations. In Table 4 we show the free energy change of the mutation process without propranolol (Mut), for which hysteresis is largest in Table 1. The free energy estimate from the forward mutation remains relatively stable, in terms of both free energy and uncertainty (19 ± 2.3 kJ/mol); although the reverse estimates of these both decrease gradually, the hysteresis still remains at 19 kJ/mol after 1 ns at every *λ* point. Interestingly, the forward simulation was repeated starting from the final configuration of the reverse process, and a very similar estimate (16.9 ± 3.5 kJ/mol) as for the first forward calculation was obtained once again. This suggests that the hysteresis does not occur because the reverse simulation leads to an alternative configuration, but rather that the sampling in the two directions is insufficiently complete. The HRE simulation of this process yields a free energy estimate between the forward and reverse simulations, which might suggest mixing of multiple conformational states appearing at different *λ* points. Figure 5 shows the free energy profiles for these processes, where the data for the reverse simulation are inverted for ease of comparison ($\lambda \to 1 - \lambda ,\,\partial {\rm H}/\partial \lambda \to - \partial {\rm H}/\partial \lambda$). Data obtained after 200, 600, and 1,000 ps per *λ* point are shown in this figure. The free energy profile of the reverse simulation converges slowly towards the profile of the forward simulation, and the HRE profile resembles the average of the two TIs as the simulation time progresses. Note that this was not the case for all simulations, as is also visible from the integrated free energy differences of Table 1, and that HRE energy profiles at high *λ* values often yielded higher ensemble averages than either of the TI (data not shown).

**Table 4:** Free energy change in a specific process

Time (ps/LP)	Δ*G*_Mut_^for^ (kJ/mol)	Δ*G*_Mut_^rev^ (kJ/mol)	Δ*G*_Mut_^hre^ (kJ/mol)
200.0	20.0 ± 2.2	4.1 ± 3.8	13.0 ± 2.3
400.0	17.6 ± 2.4	2.1 ± 3.6	10.6 ± 3.6
600.0	18.7 ± 2.3	1.7 ± 3.4	9.3 ± 2.5
800.0	19.3 ± 2.3	0.4 ± 3.3	9.0 ± 3.2
1,000.0	19.7 ± 2.4	−0.7 ± 3.2	8.9 ± 2.9

Integrated free energy of the CYP2D6 mutation process from forward and reverse thermodynamic integration, and Hamiltonian replica exchange, after a given simulation time length per *λ* point. In a hysteresis-free system forward and reverse processes should yield free energies of equal values with opposing signs

### Geometric analysis of states

To rationalize our simulation results, we collected the trajectory data for physically meaningful states (WT, WT + *R*-PPD, WT + *S*-PPD, Mut, Mut + *R*-PPD, Mut + *S*-PPD, *R*-PPD, *S*-PPD) from all TI and HRE simulations used to build the thermodynamic schemes. The trajectories belonging to the same physical state were compared to find the molecular reasons for hysteresis and for deviations from cycle closure. All trajectories belonging to the same state were subsequently pooled to obtain an ensemble average. The atom-positional-root-mean-square deviations (RMSD) of the protein backbone atoms never exceeded 0.25 nm within a given state, and the occurrence of the secondary structure elements (Kabsch and Sander 1983) and the total number of hydrogen bonds also remained very similar (data not shown). We compared snapshots from our simulation with the original crystal structure (pdb-code: 2F9Q) and with the newly released crystal structures (3QM4, 3TBG, 3TDA). The backbone RMSD values are maximally 0.16 nm, suggesting generally good structural agreement. This analysis suggests that CYP2D6 remains relatively stable during our simulations, with no significant change or drift throughout the various simulation processes.

**Fig. 6:**
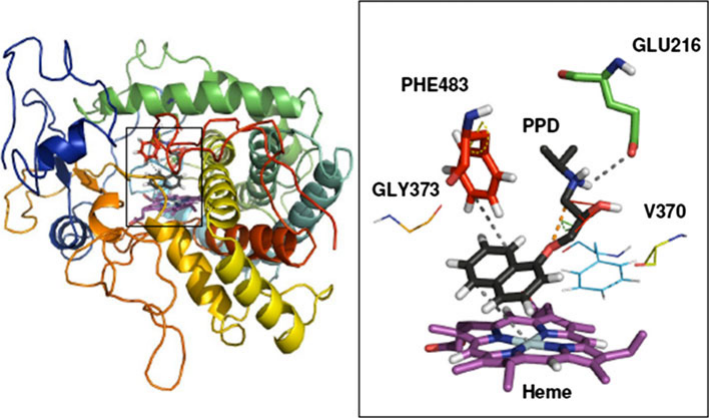
CYP2D6 active centre. The full cytochrome P450 (WT) structure is displayed on the *left hand side*, and a magnified view of the active centre is shown on the *right hand side*. Propranolol (*R*-PPD) is in *black*, heme in *purple*, and amino acids with significant interactions are shown in *colours* according their position in the sequence. *Dashed lines in grey* represent the distances measured in the geometric analysis (PHE483-PPD, PPD-GLU216, and heme-PPD). The PPD head–tail polar group distance, the chiral dihedral, and the head torsion are shown in *orange*, *red*, and *green* respectively, and the F483 ring torsion is shown in *yellow*

Figure 6 shows some of the properties in the CYP2D6 active site that were monitored explicitly over the simulations.

The improper dihedral (torsion) of the chiral carbon of PPD, and the *χ*_1_ angle of the F483 side chain were monitored, because they are closely related to the thermodynamic processes simulated. The PPD chiral improper dihedral fluctuated by ~15° around an average value of +35° (*R*-PPD), −35° (*S*-PPD), or 180° (F-PPD) depending on the simulation. The F483 side chain torsion was evenly distributed in the mutant states (where it consisted of non-interacting dummy atoms) and had a wide distribution between 50° and 200° in the wild type states, with a highly significant peak at 70° and a smaller one at approximately 150°. Note that the F483 sidechain was observed in significantly different orientations in the different crystal structures, which seems to be adequately represented by our simulations. Other properties, for example the distance between the F483 and PPD rings, the distance between the PPD ring and the heme iron, the PPD head torsion, and head–tail polar group distance were more varied throughout the simulations. In several cases one or two trajectories from a particular state resulted in completely different distributions in the visited conformations, but no obvious pattern emerged which could be identified as a key factor in the hysteresis. Replica exchange simulations usually have a wider distribution and a better agreement between trajectories from different processes compared with the TI simulations, but not in every case. When the TI (forward and reverse) and HRE trajectories of the same end state were compared, we observed three typical cases:

1. all three simulations visit a highly overlapping conformational distribution (77 %);
2. TI simulations had different distributions, and HRE overlapped with both (18 %); and
3. one of the three simulations had a significantly different distribution (low overlap; 5 %)

**Fig. 7:**
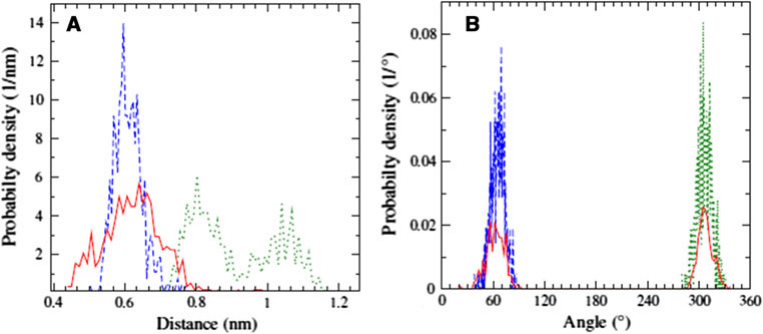
Sampling examples. Examples of distributions obtained in HRE and TI simulations in selected cases. Distributions for the same state shown for forward TI (*dashed blue*), reverse TI (*green dotted*), and replica exchange (*red solid*). **a** the F483–PPD ring distance in the WT + *R*-PPD state, where replica exchange did not sample the reverse TI conformations. **b** The PPD head torsion in the Mut_R state, where replica exchange overlaps with both (mutually distinct) TI distributions equally well

The percentages indicate how much of the geometric measurements fall in the respective categories. In more than 75 % of cases 2 and 3 replica exchange simulations visit regions in the conformational space that overlap with the distributions obtained from TI simulations. Figure 7 displays two typical examples of geometric distributions, when a difference between the three simulations occurs.

**Fig. 8:**
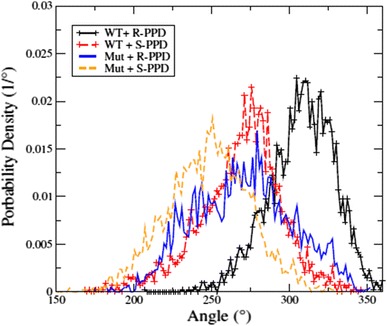
Propranolol orientation. Distribution of the angle between the norms of the PPD and heme ring systems. *Different colours* represent the distribution of orientation in the different states defined in Fig. 4

Even if not all simulations cover the same conformational space, the pooled trajectories may reveal differences between the individual states concerning the PPD position and backbone conformations. As a general trend PPD seems to be further away from the heme iron in the wild type enzyme with an average 0.58 nm distance, compared with 0.46 nm in the mutant. It is likely that the disappearance of the F483 ring gives the PPD more space to move. Approximately 5 % of the time the C4 atom of PPD gets within 0.25 nm of the heme Fe, whereas this not observed for the wild type protein. Finally we monitored the distribution of the angle between the norms of the PPD ring system compared with the plane of the heme (Fig. 8). The average angle of *R*-PPD in the wild type protein is approximately 315° whereas it is 275° for *S*-PPD. On mutation the distribution widens significantly and maxima are shifted by approximately 25° in both cases. The wider distribution again suggests more mobility in the mutant, which may also correlate with the observation that the calculated relative binding free energy of *R*-PPD and *S*-PPD in the mutant is close to zero, because a larger active site imposes fewer chiral restraints. The geometric differences may also lead to a possible change in the metabolic reaction rate for formation of the 4-hydroxypropranolol metabolite (Yoshimoto et al. 1995).

### Rationalization of affinity differences

After checking the general structure, we will rationalize the results obtained from the thermodynamic calculations. Although the total number of hydrogen bonds associated with propranolol remains the same (three hydrogen bonds on average), the partners of PPD change substantially in the different states (Table 5). *R*-PPD forms, on average, 0.5 hydrogen bonds with the protein whereas *S*-PPD forms approximately 1.5. This difference is compensated by the hydrogen bonds formed between PPD and the water molecules in the cavity, suggesting that *R*-PPD interacts via water bridges whereas *S*-PPD has more direct contact. The main hydrogen bond acceptor in all cases was glutamate 216, which is commonly indicated to be one of the key participants in ligand binding (Paine 2002; Rowland et al. 2006; de Graaf et al. 2007b; Unwalla et al. 2010). E216 interacts with *R*-PPD mostly through the positively charged NH_2_ group in the tail region, but the hydrogen bond is only present in 30 % of the simulation time. On the other hand *S*-PPD forms a very stable H-bond with the NH_2_ group, and a weaker hydrogen bond with its OH group, resulting in an average of 1.2 hydrogen bonds between the two species. Another noteworthy difference in the H-bonding of *R* and *S*-PPD is the number of hydrogen bond partners during the simulations (Table 5). The F483A mutation increases the number of partners (another hint of mobility), and the observed number of hydrogen-bond partners is larger for *S*-PPD than for *R*-PPD. An increase in possible hydrogen-bonding partners suggests more conformational freedom within the binding pocket, leading to a larger entropic contribution to the free energies. Despite the increase in the number of hydrogen-bonding partners, no additional stable hydrogen bonds were observed between the protein and propranolol.

**Table 5:** Hydrogen bonding of propranolol in different states

State	WT + *R*-PPD	WT + *S*-PPD	Mut + *R*-PPD	Mut + *S*-PPD
PPD N–H → E216	0.31	0.90	0.31	0.78
PPD OH → E216	0.10	0.39	0.0	0.29
PPD-prot	0.47	1.42	0.62	1.46
PPD-solv	2.58	1.60	2.38	1.69
PPD total	3.05	3.02	3.00	3.15
Partners	34	53	71	84

The number of hydrogen bonds between propranolol and CYP2D6 (E216 and total (PPD-prot)), propranolol and water molecules (PPD-solv), and in total (PPD total) averaged over the simulation of states described in Fig. 4. Number of hydrogen bond donor–acceptor pairs between the protein and propranolol (partners)

The conformational entropy of different states was estimated by use of the Schlitter (1993) formula for PPD and the F483 side chain. For PPD, the configurational entropy was determined by performing a translational fit for all heavy atoms before the analysis. The rotational and translational entropy was included in a second analysis, where the fit was performed on the protein backbone atoms, as was the case for the F483 side chain. Table 6 lists the conformational entropy in each state, for the F483 side chain and for PPD. In this table we also calculate the entropy change upon transfer of a specific PPD stereo isomer between CYP mutants, and the exchange of different PPDs within a given protein. The last row contains the calculated contribution to the relative free energy difference in PPD affinity (ΔΔ*G*_affinity_). The entropy calculations were performed on an equal number of snapshots from the combined trajectory of each state. Even the combined trajectories were not long enough for the system to converge in all the cases, but as the entropy increase as a function of time is very similar, the differences in conformational entropy are converging faster than the individual entropies themselves. The conformational entropy of the F483 side chain is somewhat larger in the WT + *R*-PPD state than in the WT + *S*-PPD state, whereas it is identical in both mutant states (where it is not interacting, and therefore free to rotate), leading to a −4.8 kJ/mol contribution to ΔΔ*G*_affinity_ toward *S*-PPD upon mutation The internal conformational freedom of PPD does not change much upon mutation (+0.9 kJ/mol), but when rotational and translational contributions are also considered, mutating the enzyme yields −2.6 kJ/mol entropic preference towards *S*-PPD again. The total −7.4 kJ/mol entropic contributions corresponds to the experimental and calculated free energy difference, suggesting that the driving force of stereoselectivity may be entropic rather than enthalpic in this particular case. This seems to agree with observations that a very similar number of hydrogen bonds were observed (Table 5). Although the starting structure of the simulations might affect the observed ensemble (and thus enthalpic and entropic contributions), the closing thermodynamic cycles, the generally good agreement between the structure backbones, and the fact that the F483 side chain samples conformations very similar to those observed in the different crystal structures increases confidence in our results.

**Table 6:** Calculated conformational entropy contributions

System	F483 ring (kJ/mol)	PPD (kJ/mol)	PPD tr + rot (kJ/mol)
−*T* × *S*_WT+*R*-PPD_	−75.6	−121.9	−178.2
−*T* × *S*_Mut+*R*-PPD_	−91.1	−119.4	−182.1
−*T* × *S*_WT+*S*-PPD_	−70.9	−133.2	−181.3
−*T* × *S*_Mut+*S*-PPD_	−91.2	−129.8	−187.7
−*T* × Δ*S*_mut_^R^	−15.5	2.5	−3.8
−*T* × Δ*S*_mut_^S^	−20.3	3.4	−6.4
−*T* × Δ*S*_bind_^wt^	4.7	−11.3	−3.0
−*T* × Δ*S*_bind_^M^	−0.1	−10.4	−5.6
−*T* × ΔΔ*S*_affinity_	−4.8	0.9	−2.6

Contribution of the conformational entropy to the free energy in a given state was calculated for the F483 side chain (F483 ring), propranolol (PPD), and for propranolol including translational and rotational motion relative to the protein (PPD tr + rot). From the individual contributions in each state, the contributions of *R*-PPD or *S*-PPD binding (−*T* × Δ*S*_bind_), and of the mutation process with the respective PPDs bound (−*T* × Δ*S*_mut_) can be calculated. Finally the free energy contribution of conformational entropies to the relative difference in affinities (−*T* × ΔΔ*S*_affinity_) is calculated

## Conclusions

A set of thermodynamic integration (TI), and Hamiltonian replica exchange TI simulations were performed on wild type and F483A mutant versions of cytochrome P450 2D6 with *R* and *S*-propranolol, in an attempt to improve our previously poorly converged study (de Graaf et al. 2007a). The simulations were performed with a slightly improved version of the original force field (45A4), and were started from a simulation snapshot based on the crystal structure of CYP2D6 rather than a homology model. In addition, the simulation time was more than five times longer. Convergence was monitored in terms of the deviation from cycle closure and hysteresis. Prolonged TI simulations have shown increased internal consistency and a significant reduction—but not elimination—of hysteresis and deviations of cycle closure, and the calculated free energy differences scatter substantially compared with the experimentally derived values. Utilizing Hamiltonian replica exchange resolved the thermodynamic discrepancies even after 200 ps per *λ* point of simulation, and further prolonged simulation times led to reduced statistical uncertainty and more converged thermodynamic results in agreement with experiments. Geometric analysis of the trajectories collected for each state revealed that at least 600 ps per *λ* point of simulation was needed to reduce fluctuations, and that the replica exchange simulations could significantly reduce discrepancies in the sampled distributions. Altogether, Hamiltonian replica exchange proved to be a useful tool to ensure internal consistency of the thermodynamic data. However, the geometric analysis proved that complex systems such as CYP2D6 and propranolol might require even longer simulation time in addition to improved sampling techniques to provide proper conformational distributions. The rationalization based on pooled simulation data did not reveal significant differences in enthalpic contributions to the free energy differences, but a changed mobility of the ligand molecule suggests mostly entropic contributions to differences between binding affinities.
